# Supplementary material for: Empirically calibrated simulations reveal the limits of phenotypic clustering algorithms for biodiversity assessment in data-scarce crops
Source: PLoS One. 2025 Dec 17;20(12):e0329254. doi: 10.1371/journal.pone.0329254 (PMC12711051; doi:10.1371/journal.pone.0329254)
Supplement: S1 File — This compressed archive contains Figures S1–S12 and Table S1. (ZIP) [file pone.0329254.s001.zip › SupportingInformation/Table S1.docx]

**Table S1. Algorithmic settings and reproducibility details.**

Summary of the R functions, package versions, initialization procedures, and key parameters used for the eleven clustering algorithms benchmarked in this study. All methods were applied to z-score standardized traits with identical replication and seed control. Convergence followed the default internal criteria of the respective R functions. Code and session information are available on Zenodo (DOI: <https://doi.org/10.5281/zenodo.15877863>).

| Algorithm | R package (version) | Function(s) | k (clusters) | Initialization / Seed | Key parameters | Notes |
| --- | --- | --- | --- | --- | --- | --- |
| K-means | stats (4.3.0) | kmeans() | 3 | nstart=25; fixed seed | algorithm="Hartigan-Wong" | Multiple starts for stability |
| PAM | cluster (2.1.6) | pam() | 3 | deterministic; fixed seed | — | Medoid-based robustness |
| Ward.D2 | stats (4.3.0) | hclust() + cutree() | 3 | deterministic | method="ward.D2" | Compact cluster bias |
| GMM | mclust (6.1.1) | Mclust(G=k) | 3 | fixed seed | G=3; model selection = default | Automatic model selection |
| Spectral | kernlab (0.9-33) | specc(centers=k) | 3 | fixed seed | kernel=RBF; σ=auto | Default bandwidth heuristic |
| Affinity Propagation | apcluster (1.4.13) | negDistMat() + apcluster() | — | fixed seed | r=2; preference=default | Distance-based similarity |
| DBSCAN | dbscan (1.2.2) | kNNdist() + dbscan() | — | fixed seed | minPts=5; eps=Q0.90(kNNdist) | Data-driven heuristic |
| HDBSCAN | dbscan (1.2.2) | hdbscan() | — | fixed seed | minPts=5 | Hierarchical density clustering |
| Fuzzy C-means | e1071 (1.7-16) | cmeans(centers=k) | 3 | fixed seed | m=2 | Soft membership approach |
| TwoStep | tclust (2.1.2) | tclust(k) | 3 | fixed seed | alpha=0.05 | Robust trimming |
| SOM | kohonen (3.0.12) | som() | 3 | fixed seed | grid=k×1; rlen=100 | Neural network clustering |

*Notes: All clustering algorithms were applied to z-score standardized trait data using the same preprocessing and replication scheme. The same wrapper function, apply_clustering(), was used for execution and evaluation of all methods. Random seeds were fixed for full reproducibility. All convergence criteria correspond to default internal stopping rules of the respective R functions.*
