# Supplementary figures and images for: Empirically calibrated simulations reveal the limits of phenotypic clustering algorithms for biodiversity assessment in data-scarce crops
Source: PLoS One. 2025 Dec 17;20(12):e0329254. doi: 10.1371/journal.pone.0329254 (PMC12711051; doi:10.1371/journal.pone.0329254)

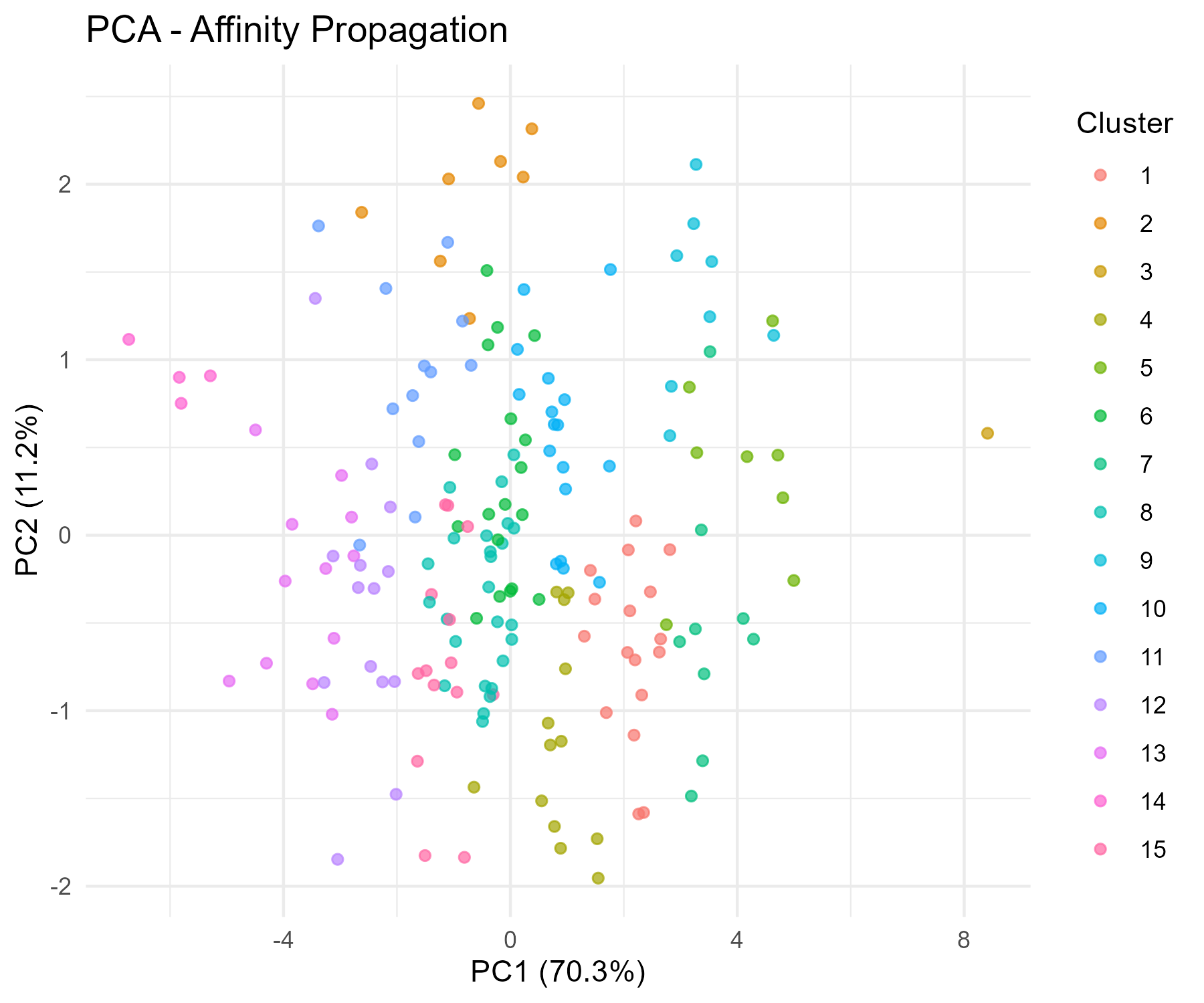

Supplement: S1 File — This compressed archive contains Figures S1–S12 and Table S1. (ZIP) [file pone.0329254.s001.zip › SupportingInformation/S1 (a).png]

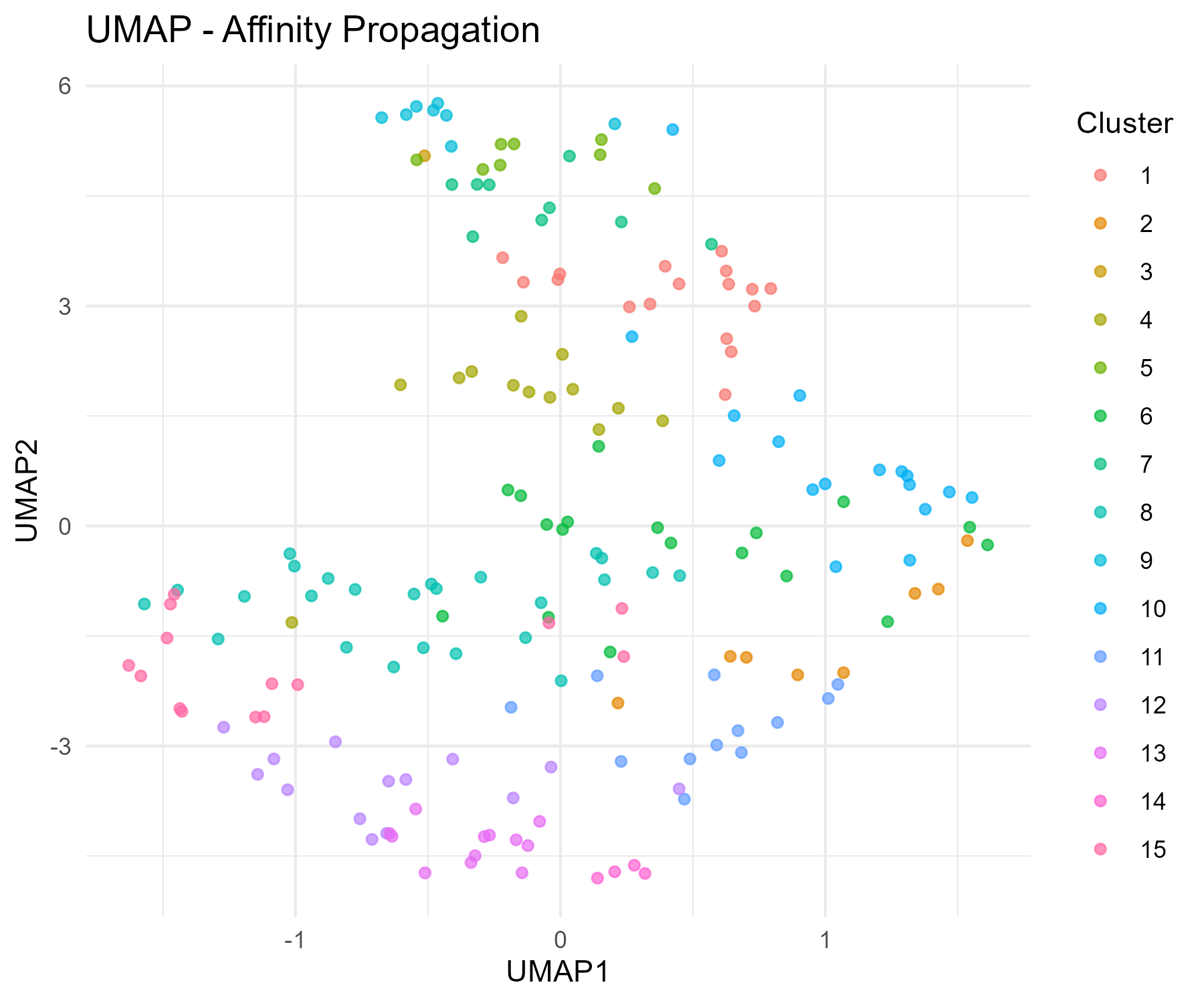

Supplement: S1 File — This compressed archive contains Figures S1–S12 and Table S1. (ZIP) [file pone.0329254.s001.zip › SupportingInformation/S1 (b).png]

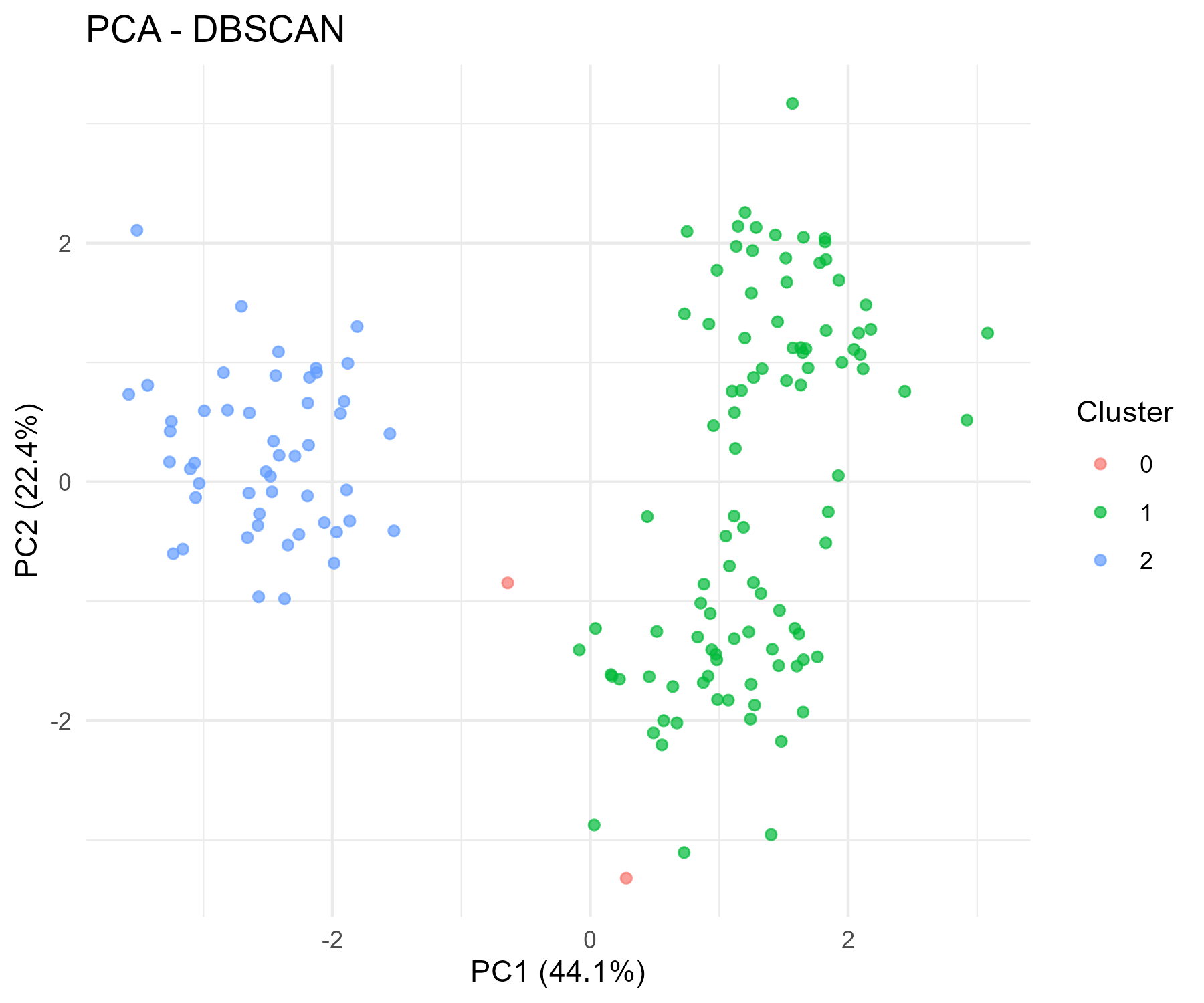

Supplement: S1 File — This compressed archive contains Figures S1–S12 and Table S1. (ZIP) [file pone.0329254.s001.zip › SupportingInformation/S10 (a).png]

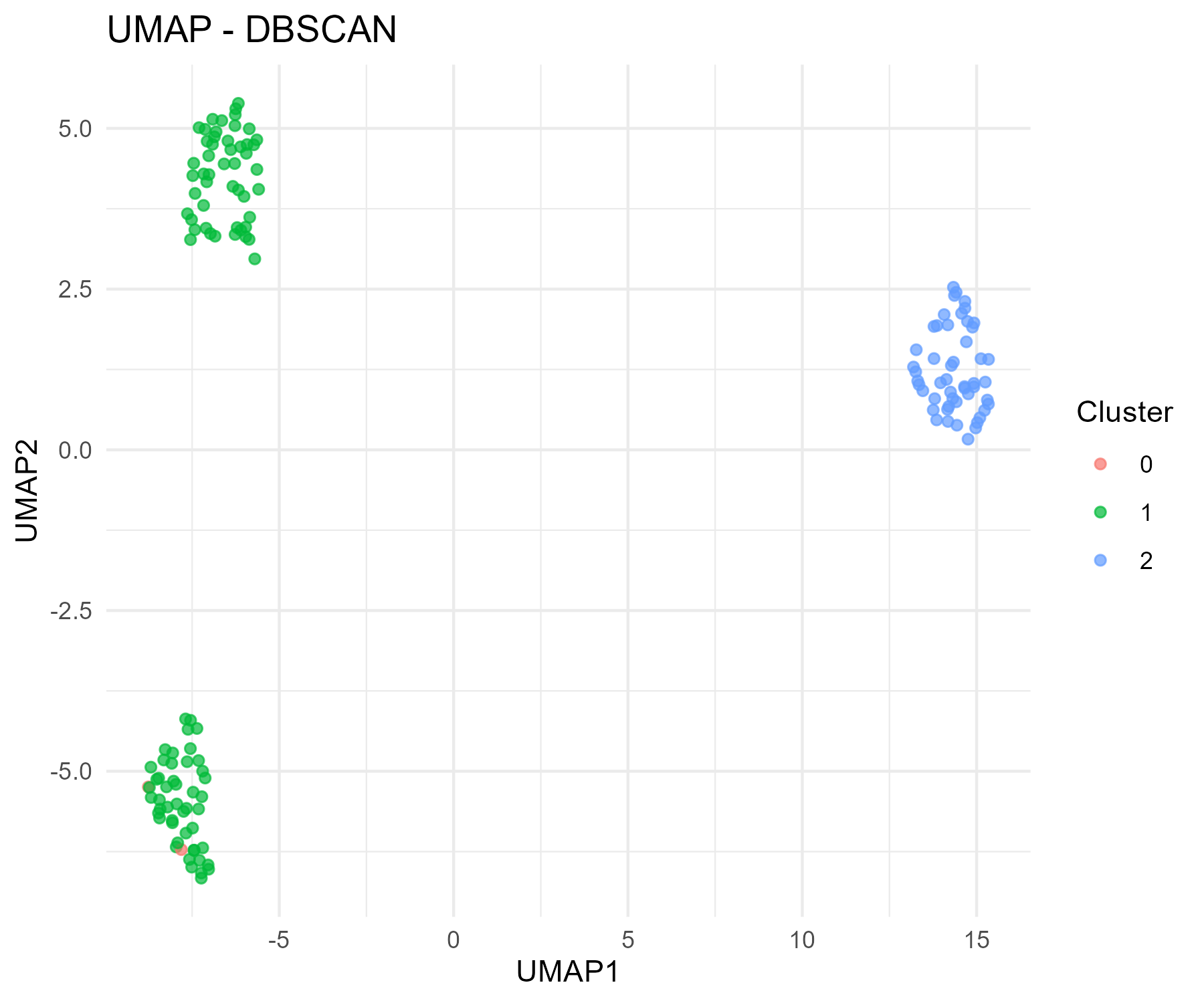

Supplement: S1 File — This compressed archive contains Figures S1–S12 and Table S1. (ZIP) [file pone.0329254.s001.zip › SupportingInformation/S10 (b).png]

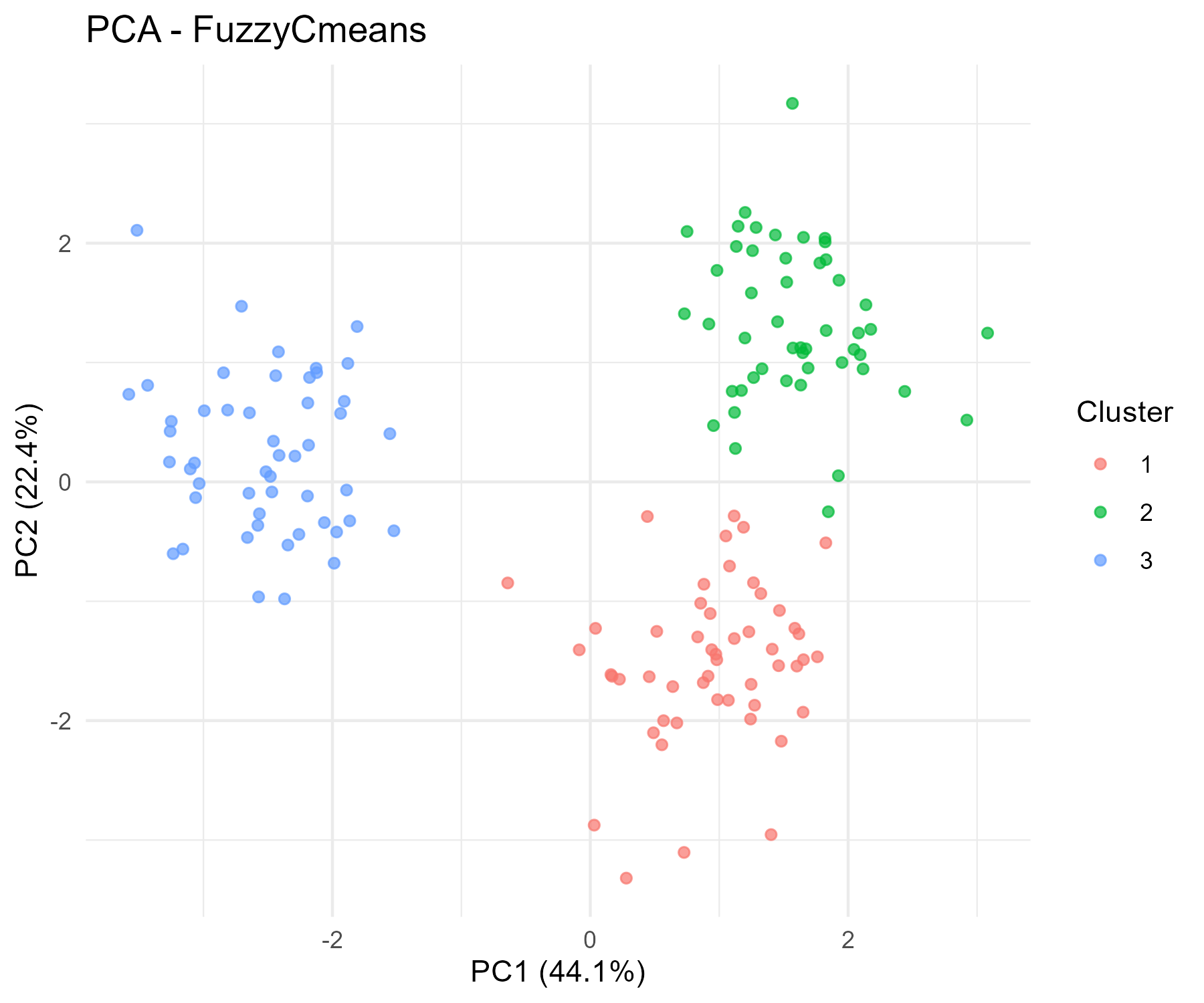

Supplement: S1 File — This compressed archive contains Figures S1–S12 and Table S1. (ZIP) [file pone.0329254.s001.zip › SupportingInformation/S11 (a).png]

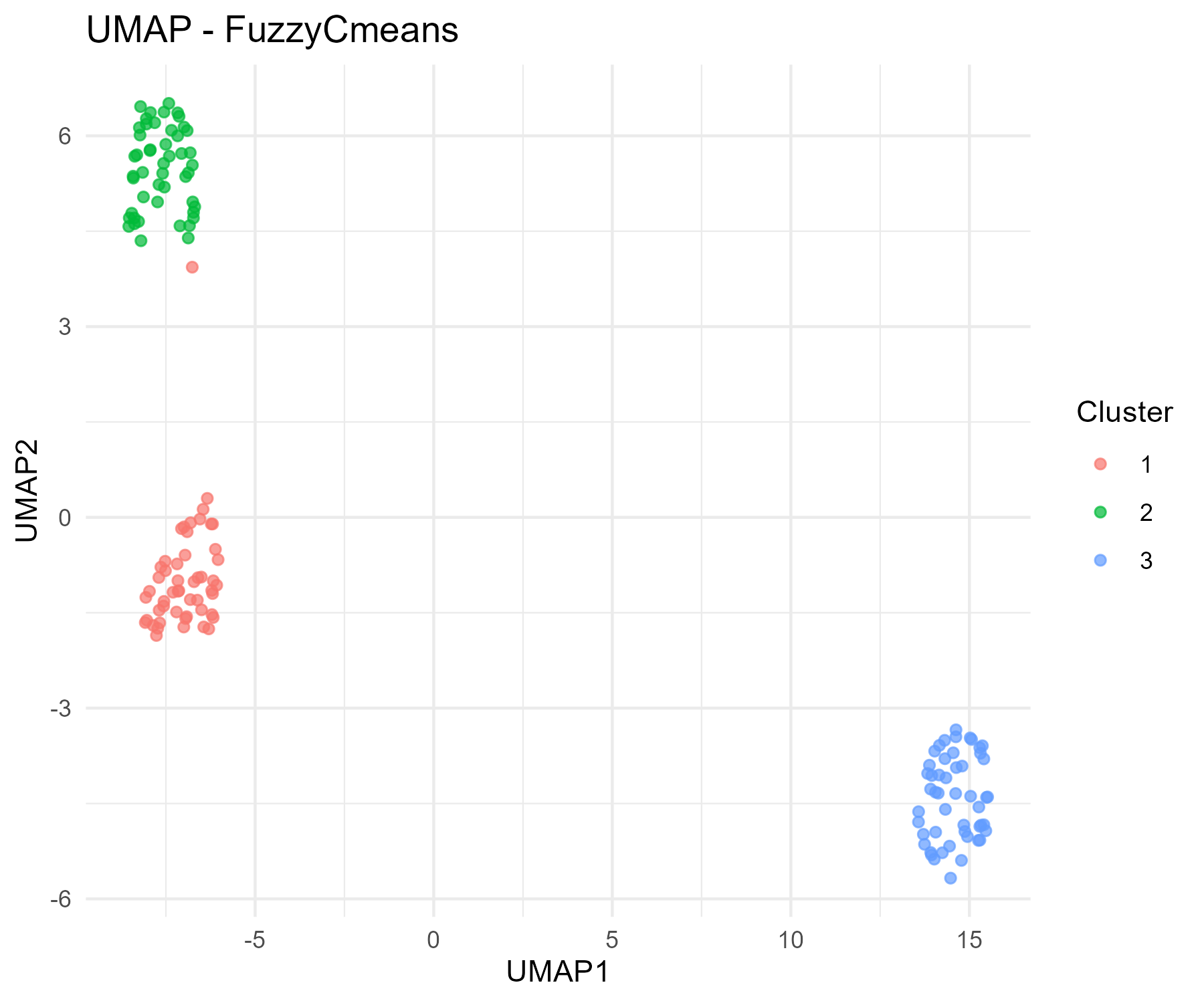

Supplement: S1 File — This compressed archive contains Figures S1–S12 and Table S1. (ZIP) [file pone.0329254.s001.zip › SupportingInformation/S11 (b).png]

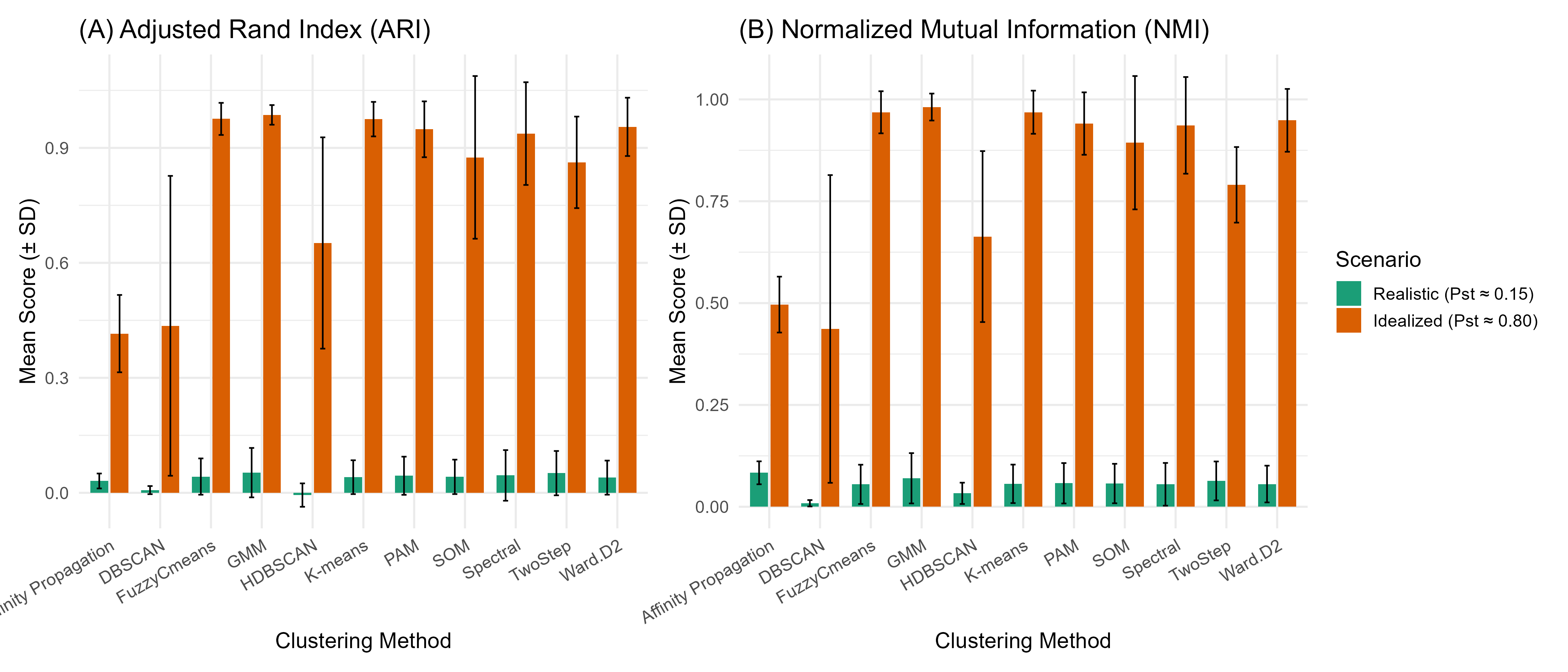

Supplement: S1 File — This compressed archive contains Figures S1–S12 and Table S1. (ZIP) [file pone.0329254.s001.zip › SupportingInformation/S12.png]

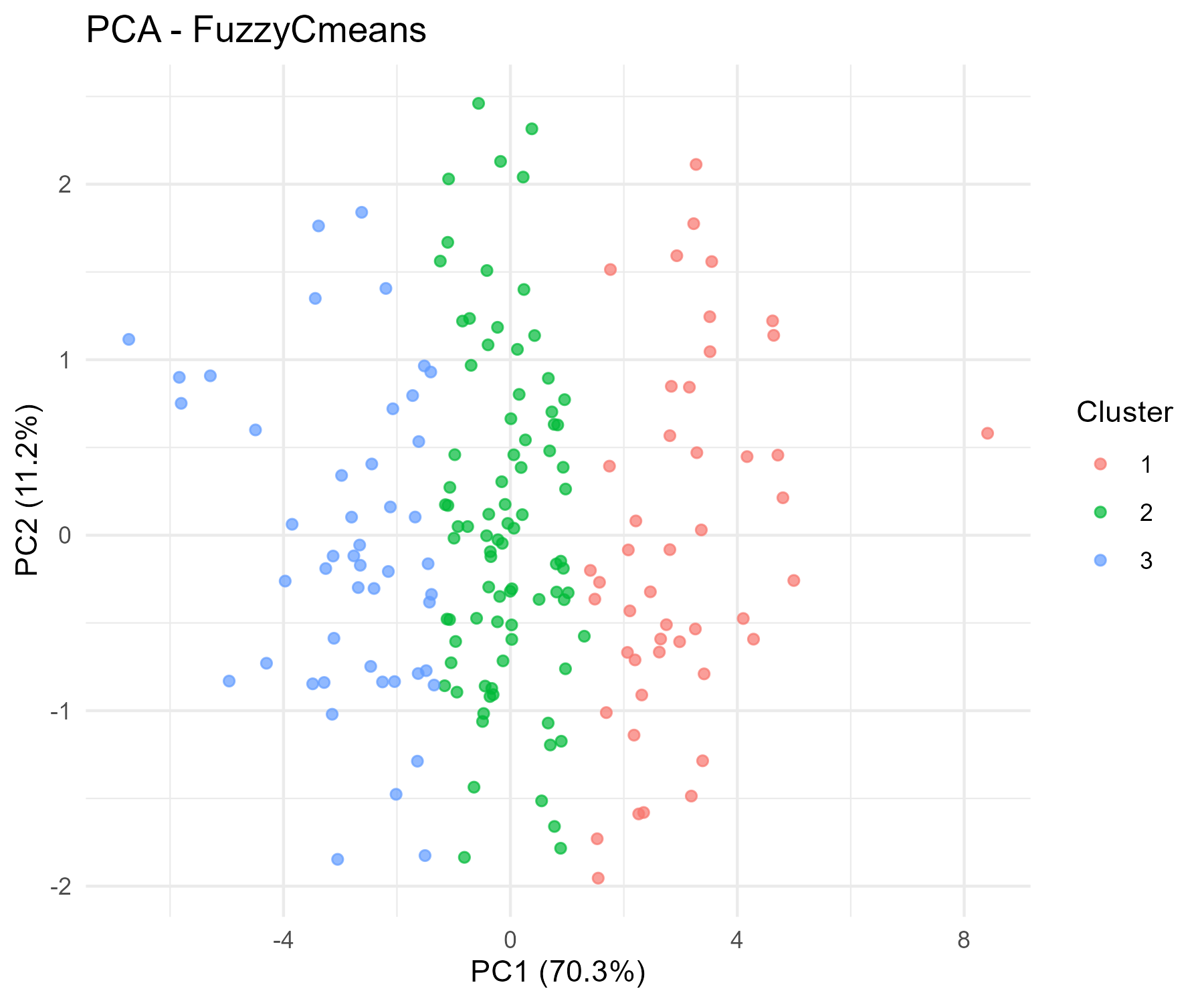

Supplement: S1 File — This compressed archive contains Figures S1–S12 and Table S1. (ZIP) [file pone.0329254.s001.zip › SupportingInformation/S2 (a).png]

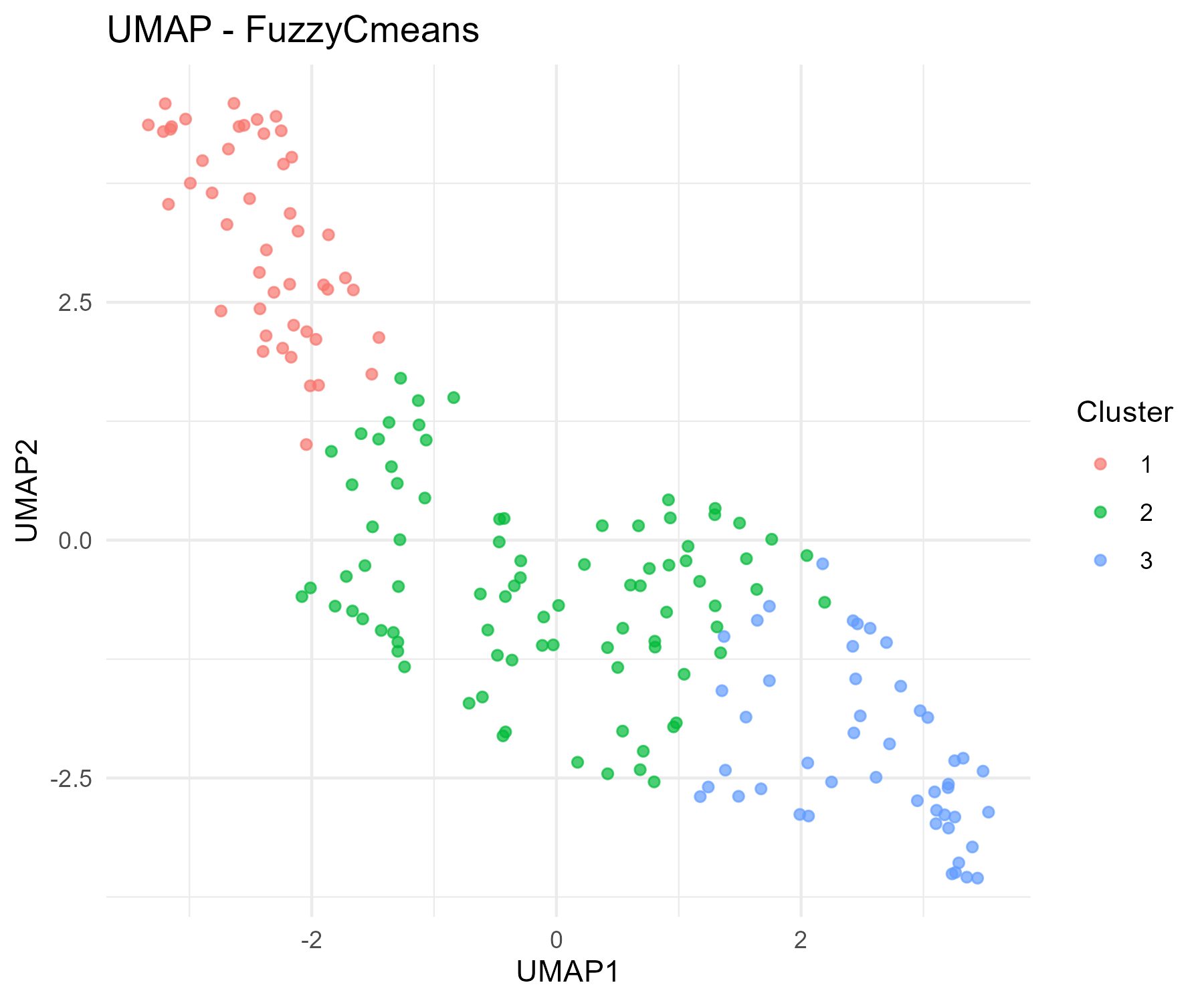

Supplement: S1 File — This compressed archive contains Figures S1–S12 and Table S1. (ZIP) [file pone.0329254.s001.zip › SupportingInformation/S2 (b).png]

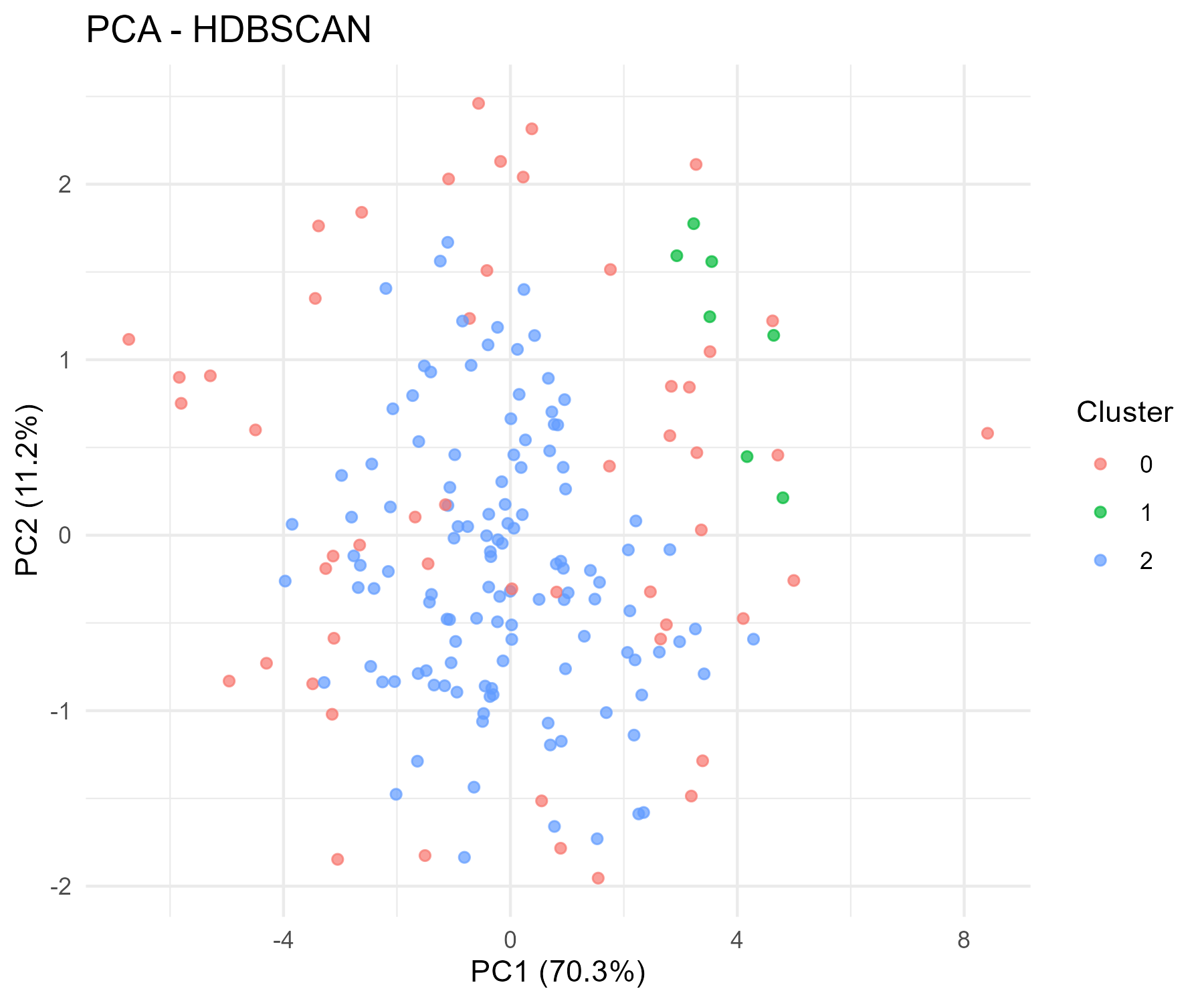

Supplement: S1 File — This compressed archive contains Figures S1–S12 and Table S1. (ZIP) [file pone.0329254.s001.zip › SupportingInformation/S3 (a).png]

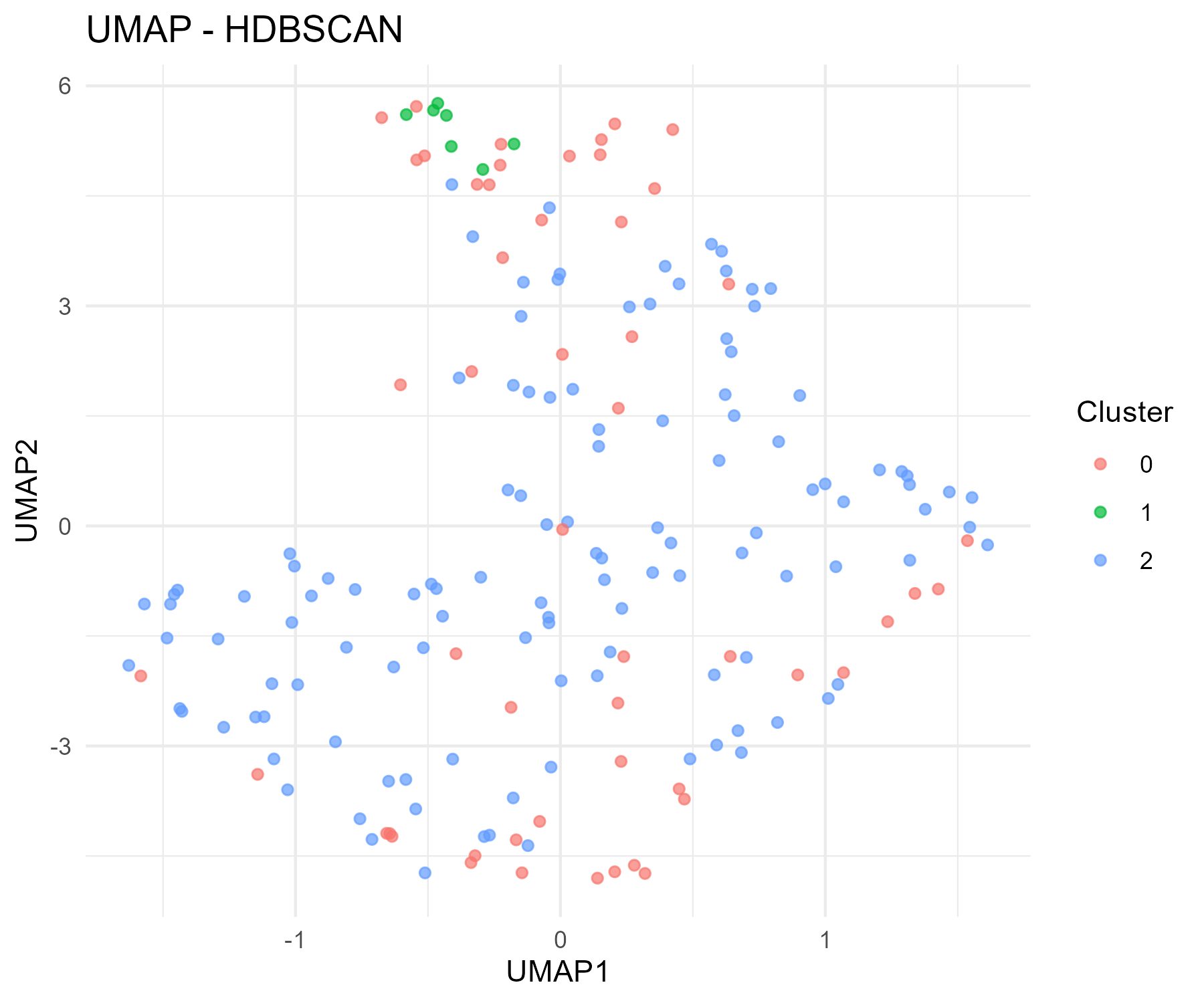

Supplement: S1 File — This compressed archive contains Figures S1–S12 and Table S1. (ZIP) [file pone.0329254.s001.zip › SupportingInformation/s3 (b).png]

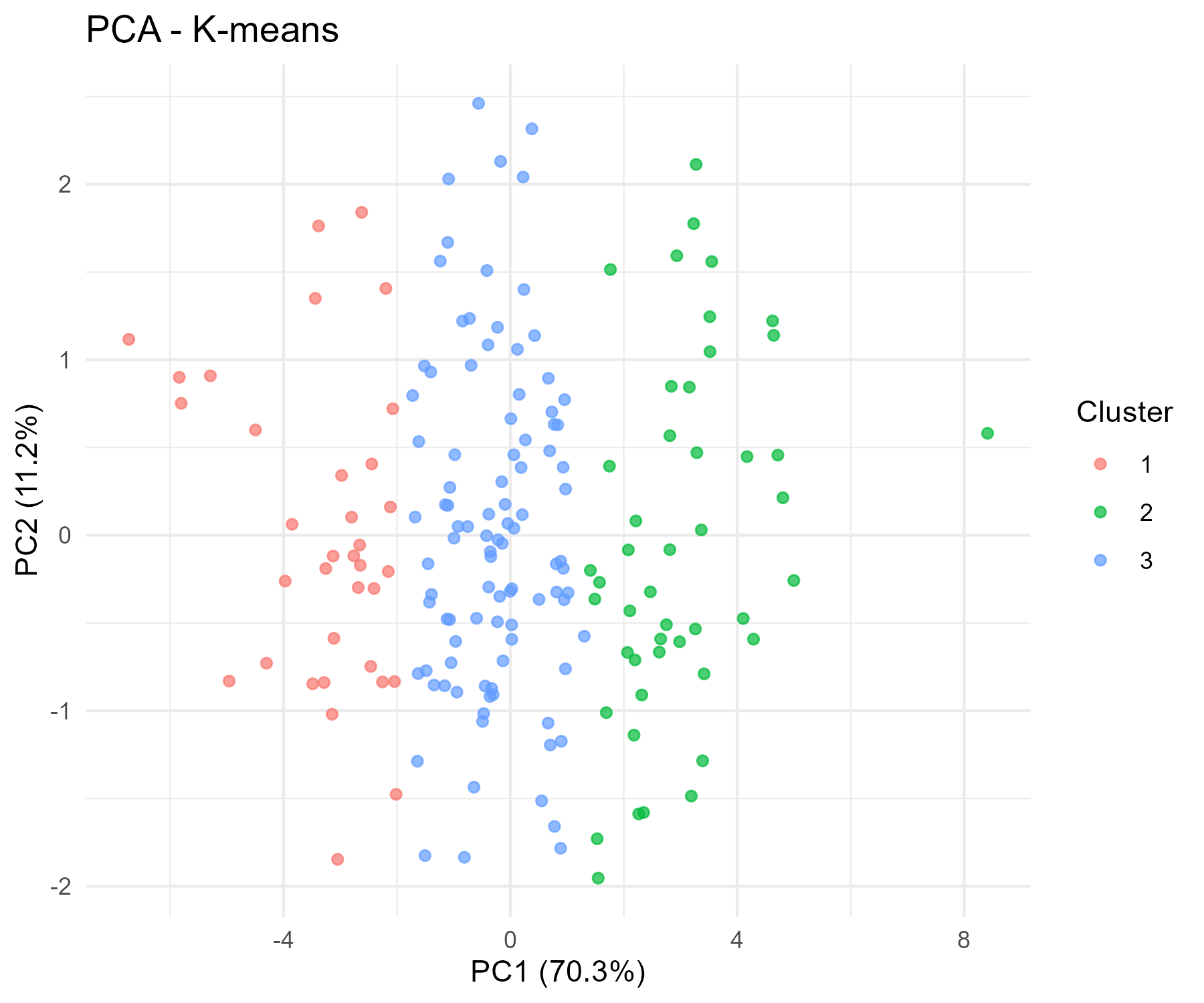

Supplement: S1 File — This compressed archive contains Figures S1–S12 and Table S1. (ZIP) [file pone.0329254.s001.zip › SupportingInformation/S4 (a).png]

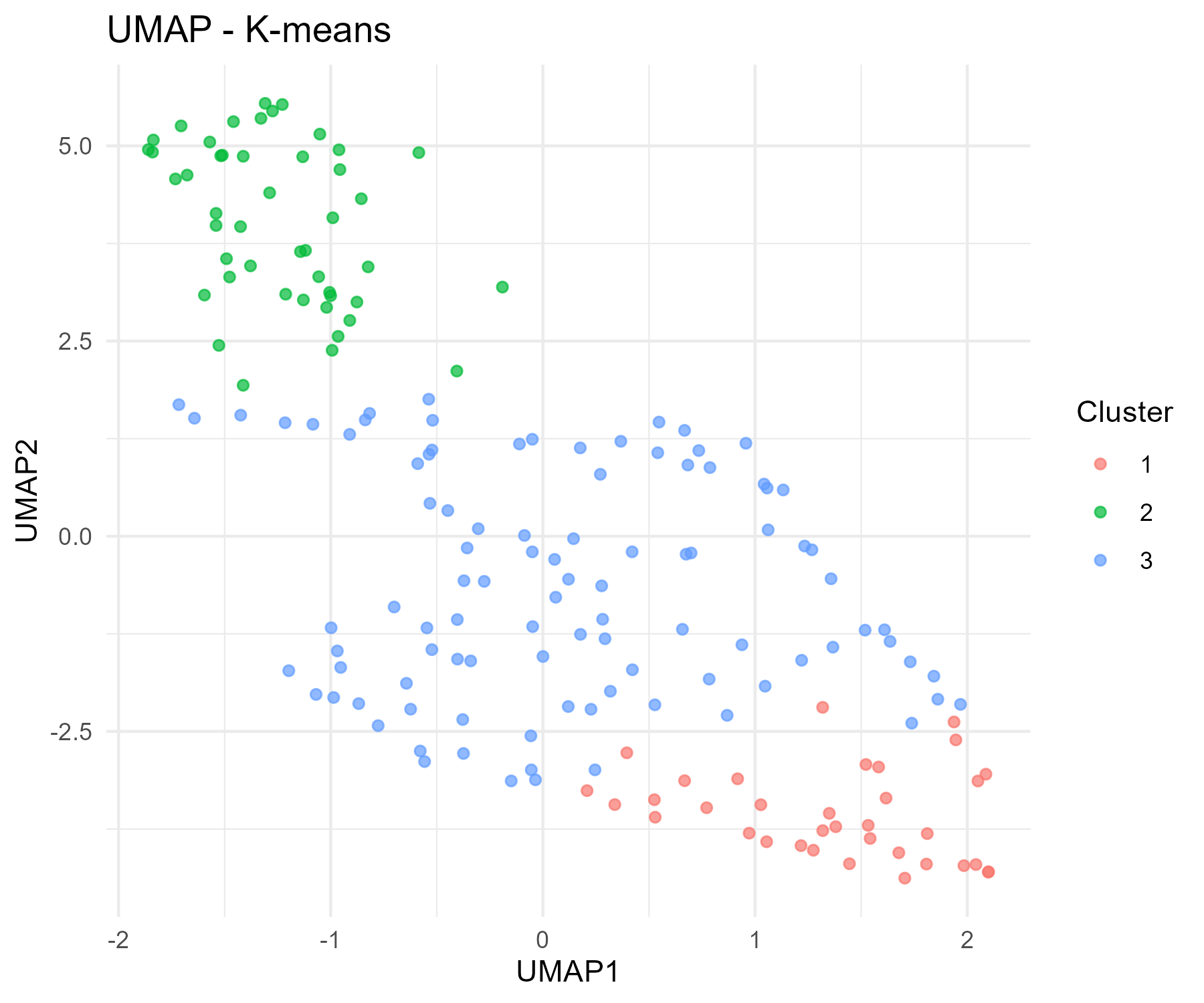

Supplement: S1 File — This compressed archive contains Figures S1–S12 and Table S1. (ZIP) [file pone.0329254.s001.zip › SupportingInformation/S4 (b).png]

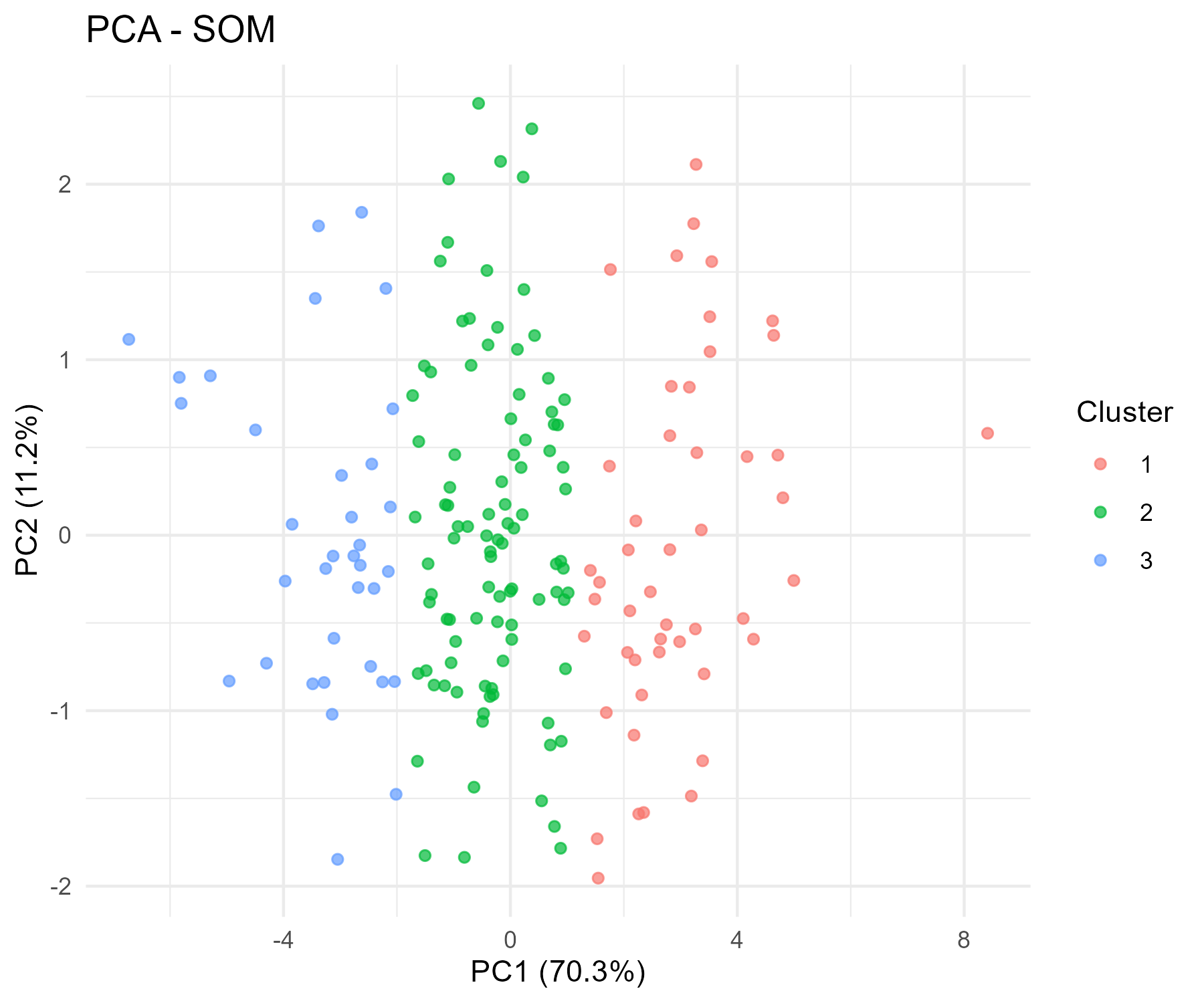

Supplement: S1 File — This compressed archive contains Figures S1–S12 and Table S1. (ZIP) [file pone.0329254.s001.zip › SupportingInformation/S5 (a).png]

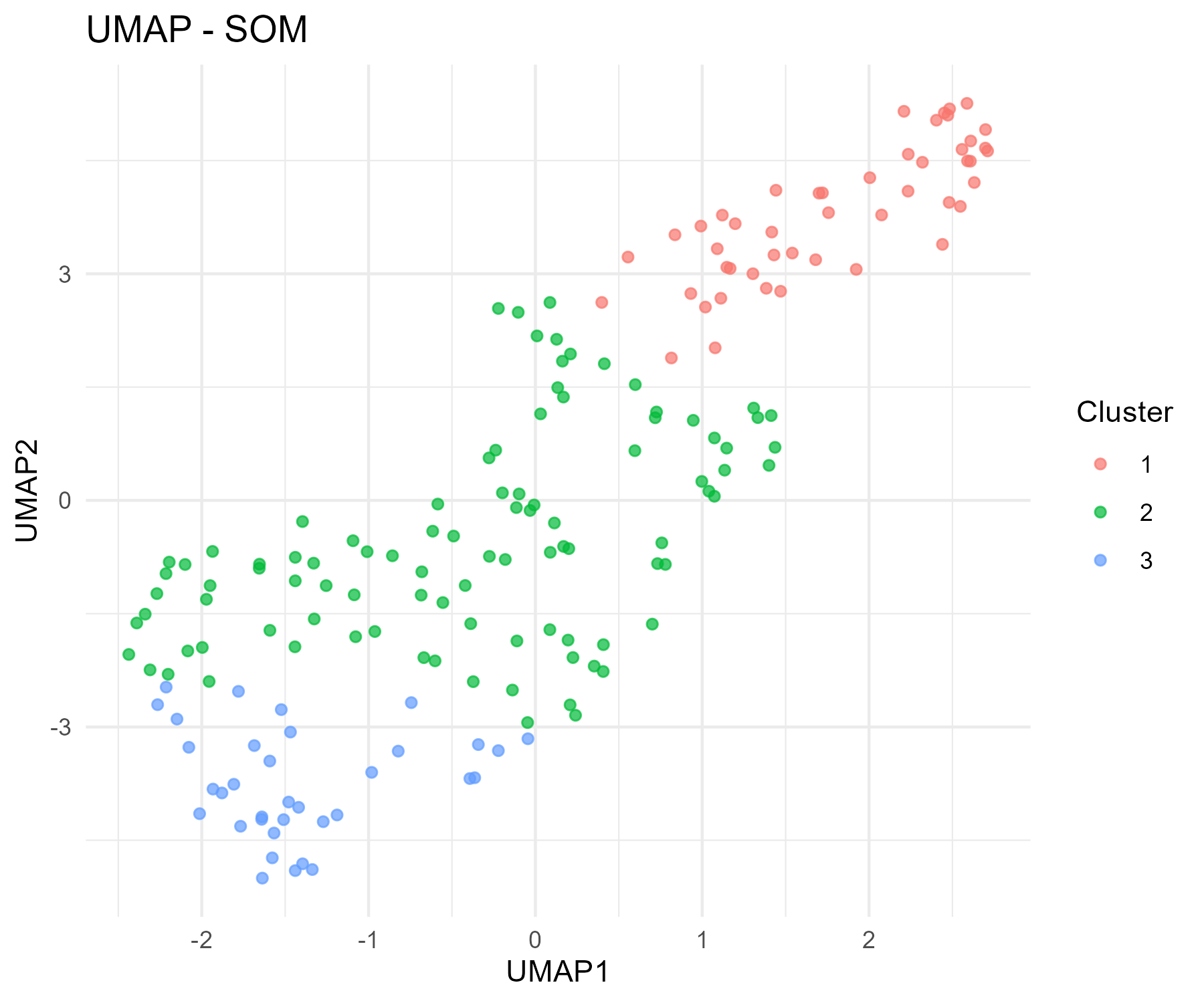

Supplement: S1 File — This compressed archive contains Figures S1–S12 and Table S1. (ZIP) [file pone.0329254.s001.zip › SupportingInformation/S5 (b).png]

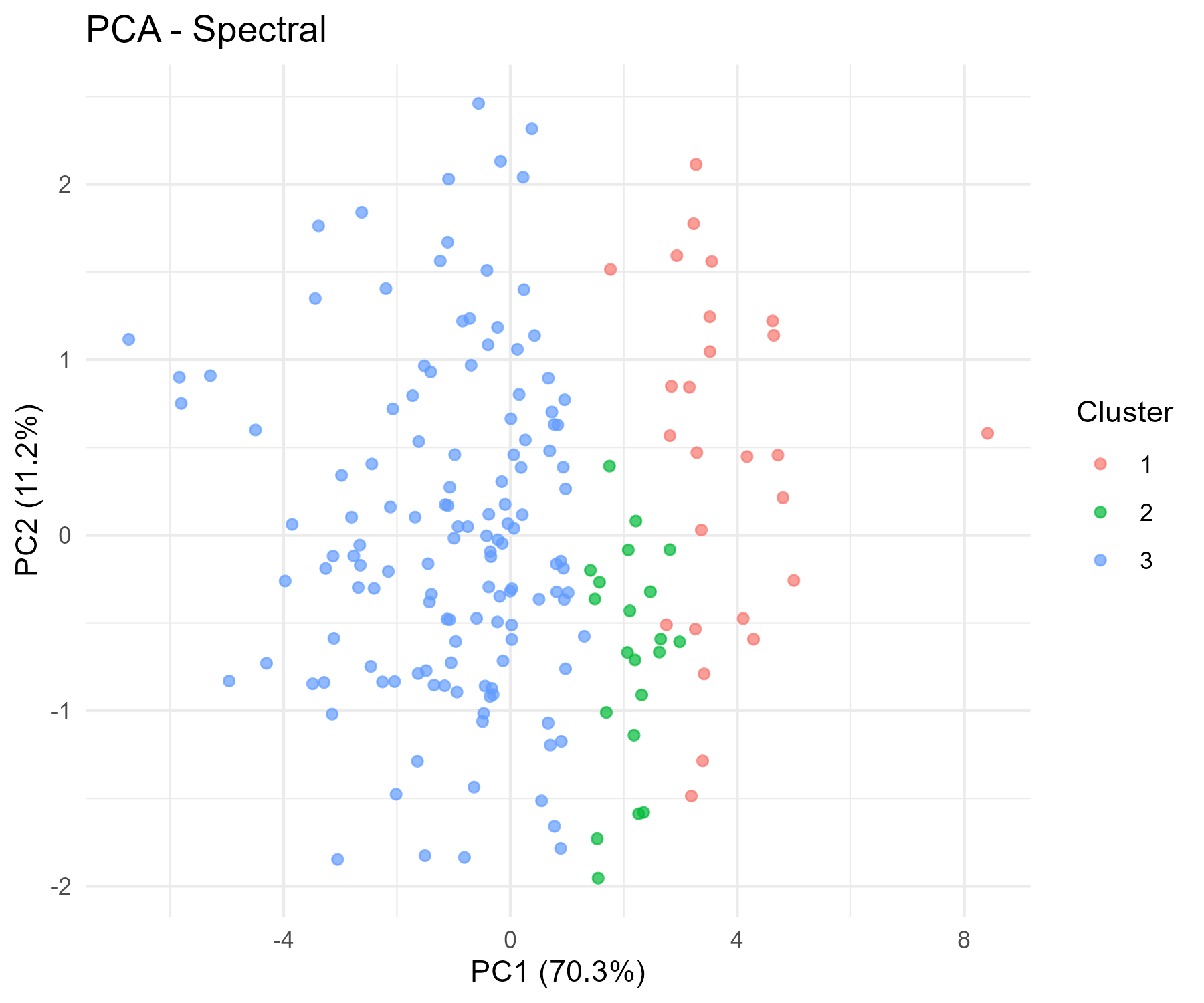

Supplement: S1 File — This compressed archive contains Figures S1–S12 and Table S1. (ZIP) [file pone.0329254.s001.zip › SupportingInformation/S6 (a).png]

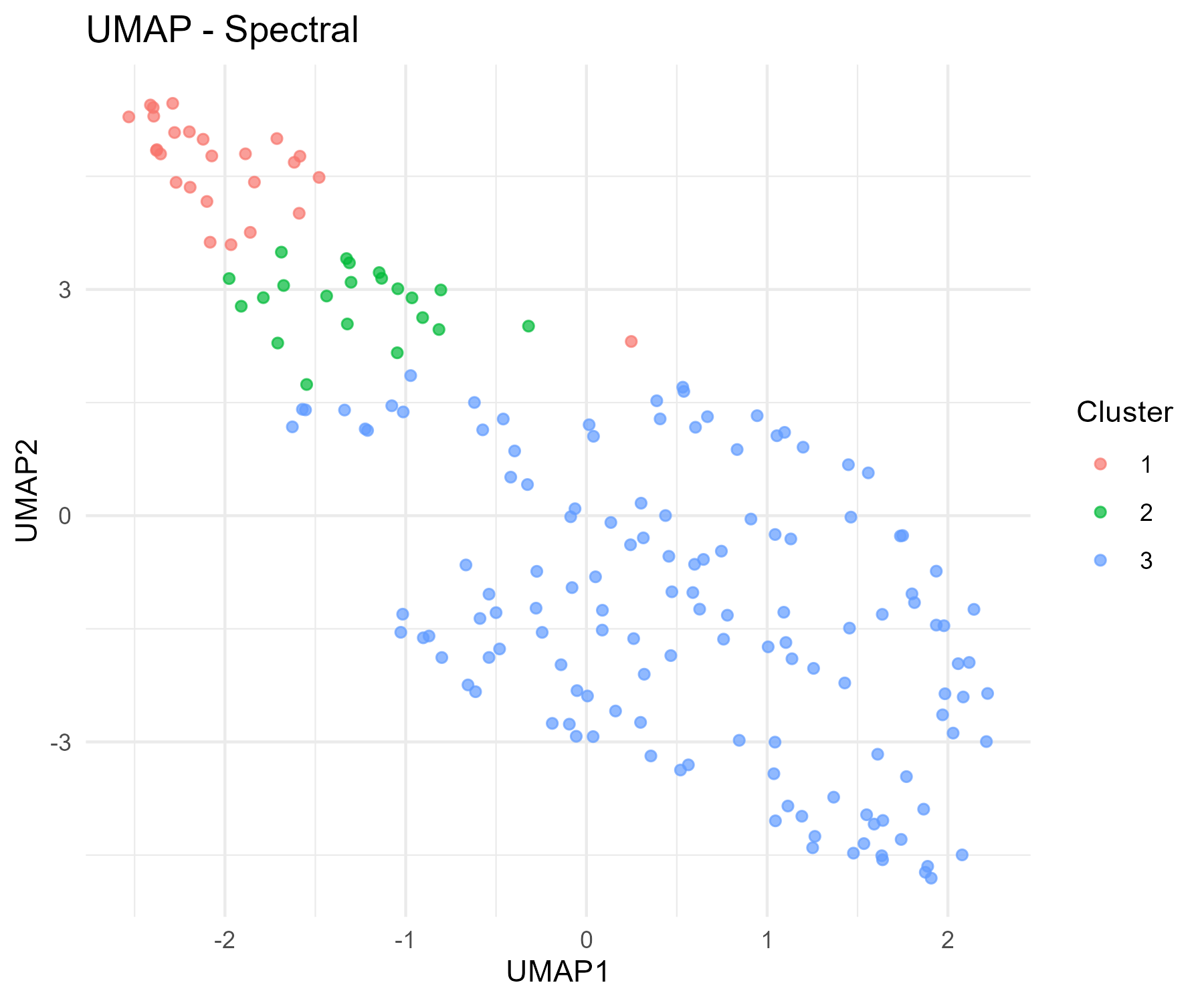

Supplement: S1 File — This compressed archive contains Figures S1–S12 and Table S1. (ZIP) [file pone.0329254.s001.zip › SupportingInformation/S6 (b).png]

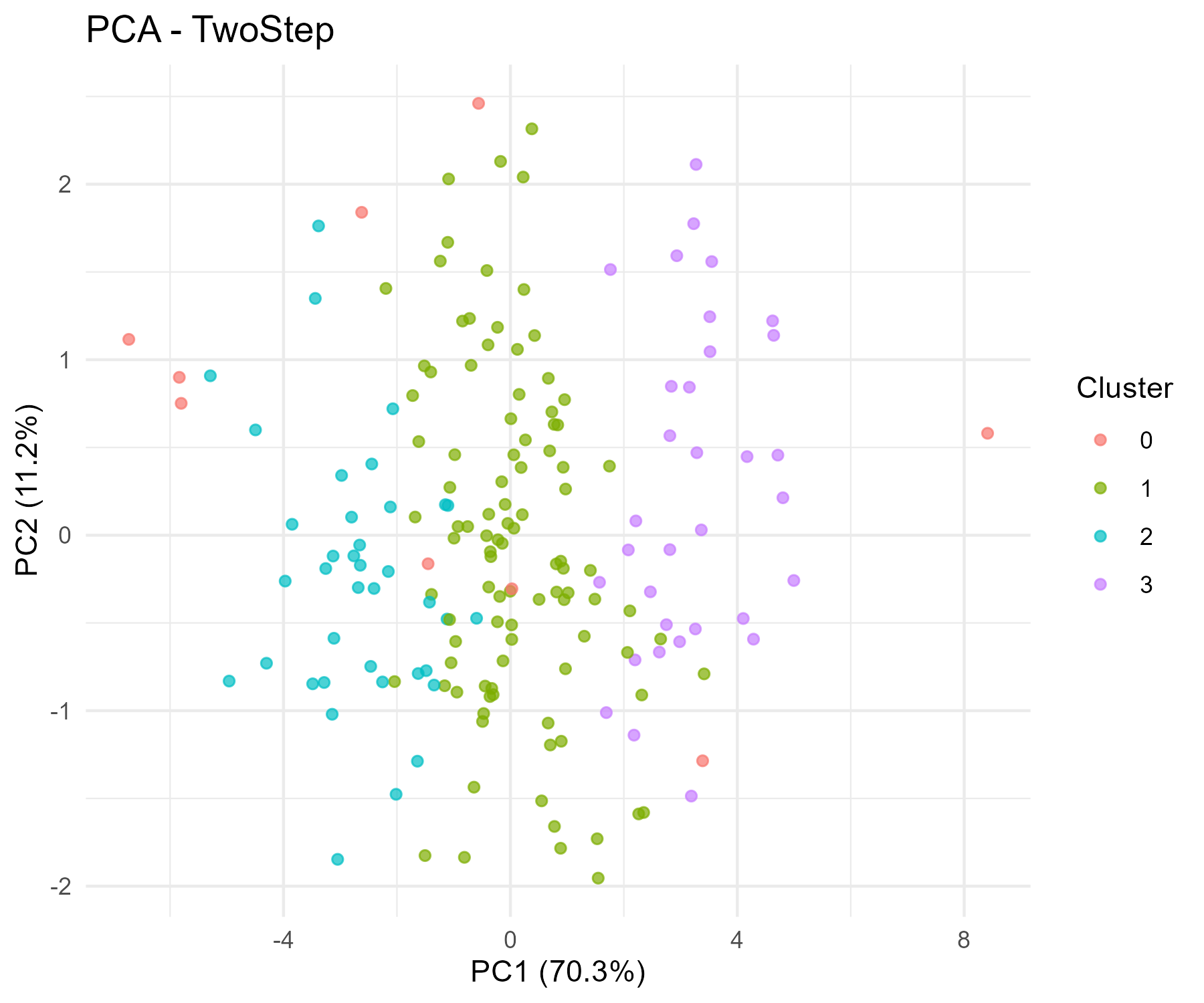

Supplement: S1 File — This compressed archive contains Figures S1–S12 and Table S1. (ZIP) [file pone.0329254.s001.zip › SupportingInformation/S7 (a).png]

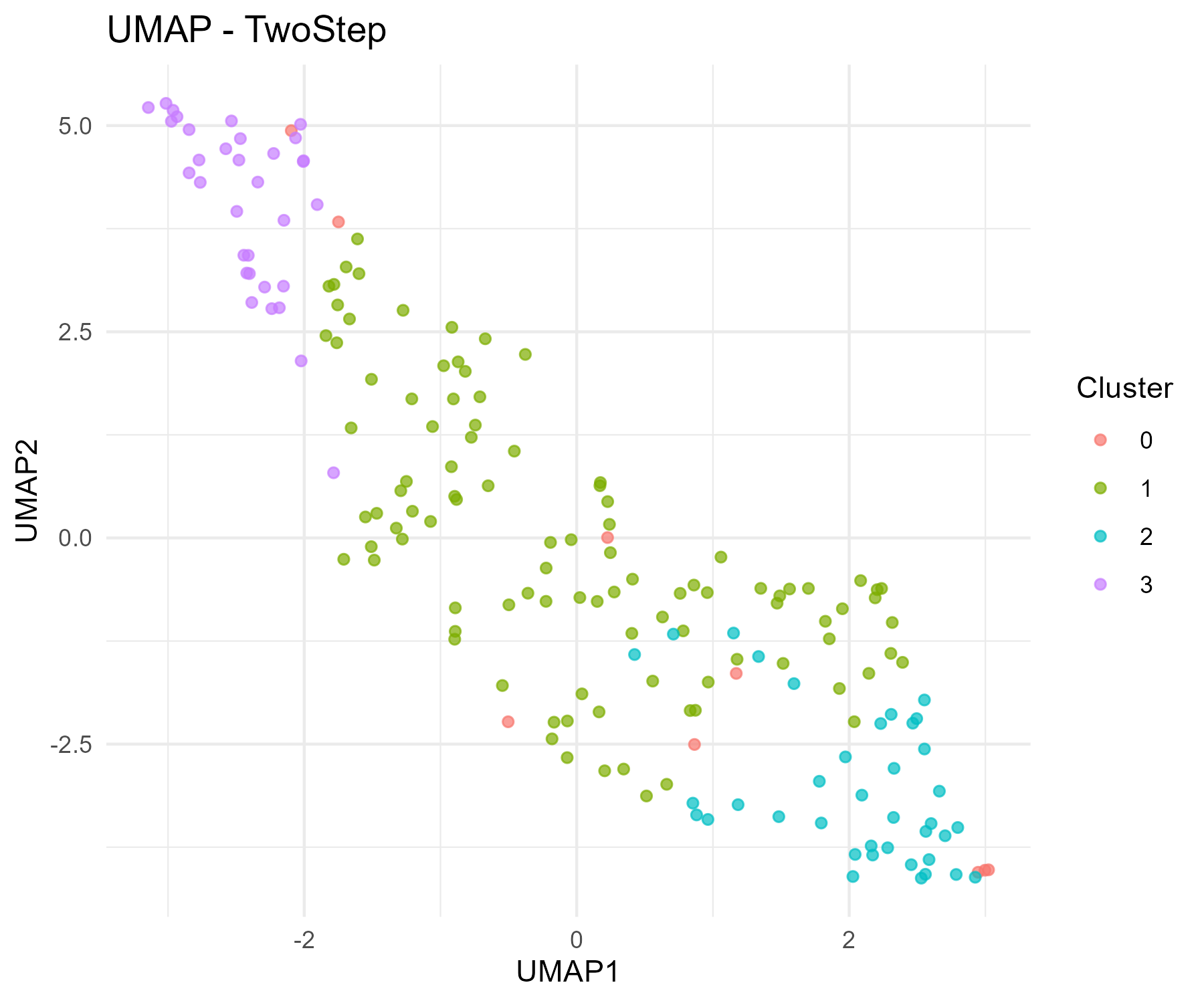

Supplement: S1 File — This compressed archive contains Figures S1–S12 and Table S1. (ZIP) [file pone.0329254.s001.zip › SupportingInformation/S7 (b).png]

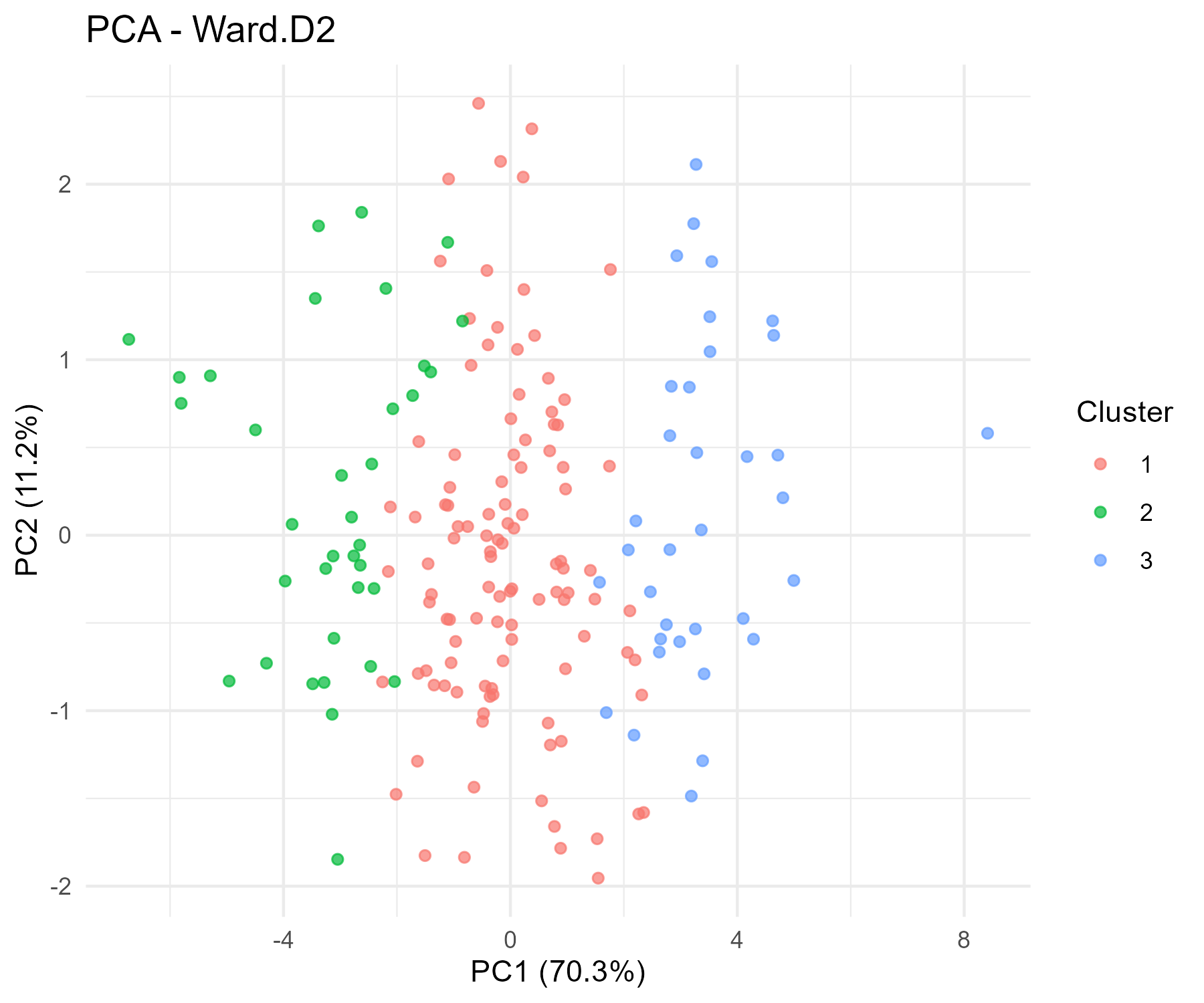

Supplement: S1 File — This compressed archive contains Figures S1–S12 and Table S1. (ZIP) [file pone.0329254.s001.zip › SupportingInformation/S8 (a).png]

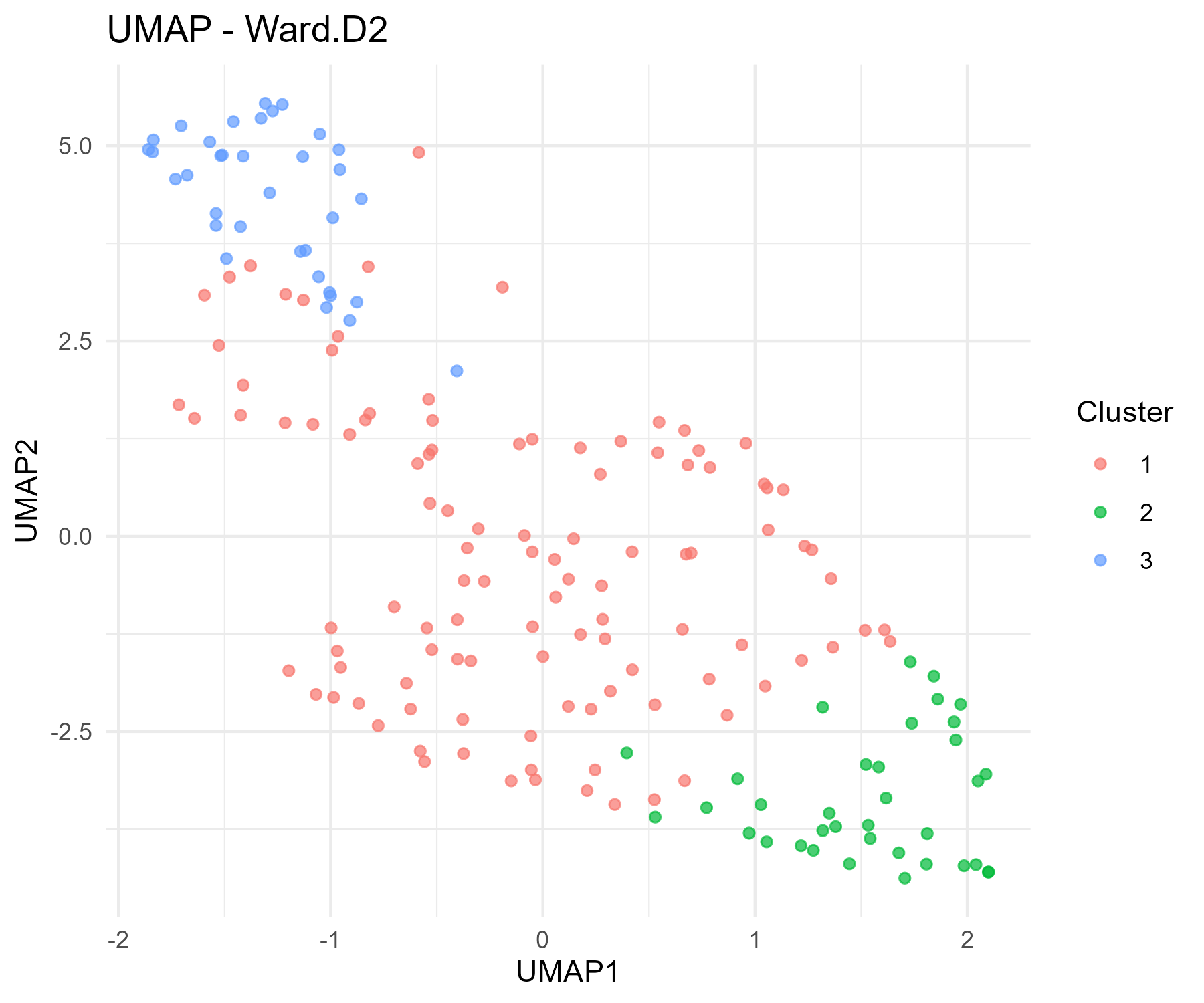

Supplement: S1 File — This compressed archive contains Figures S1–S12 and Table S1. (ZIP) [file pone.0329254.s001.zip › SupportingInformation/S8 (b).png]

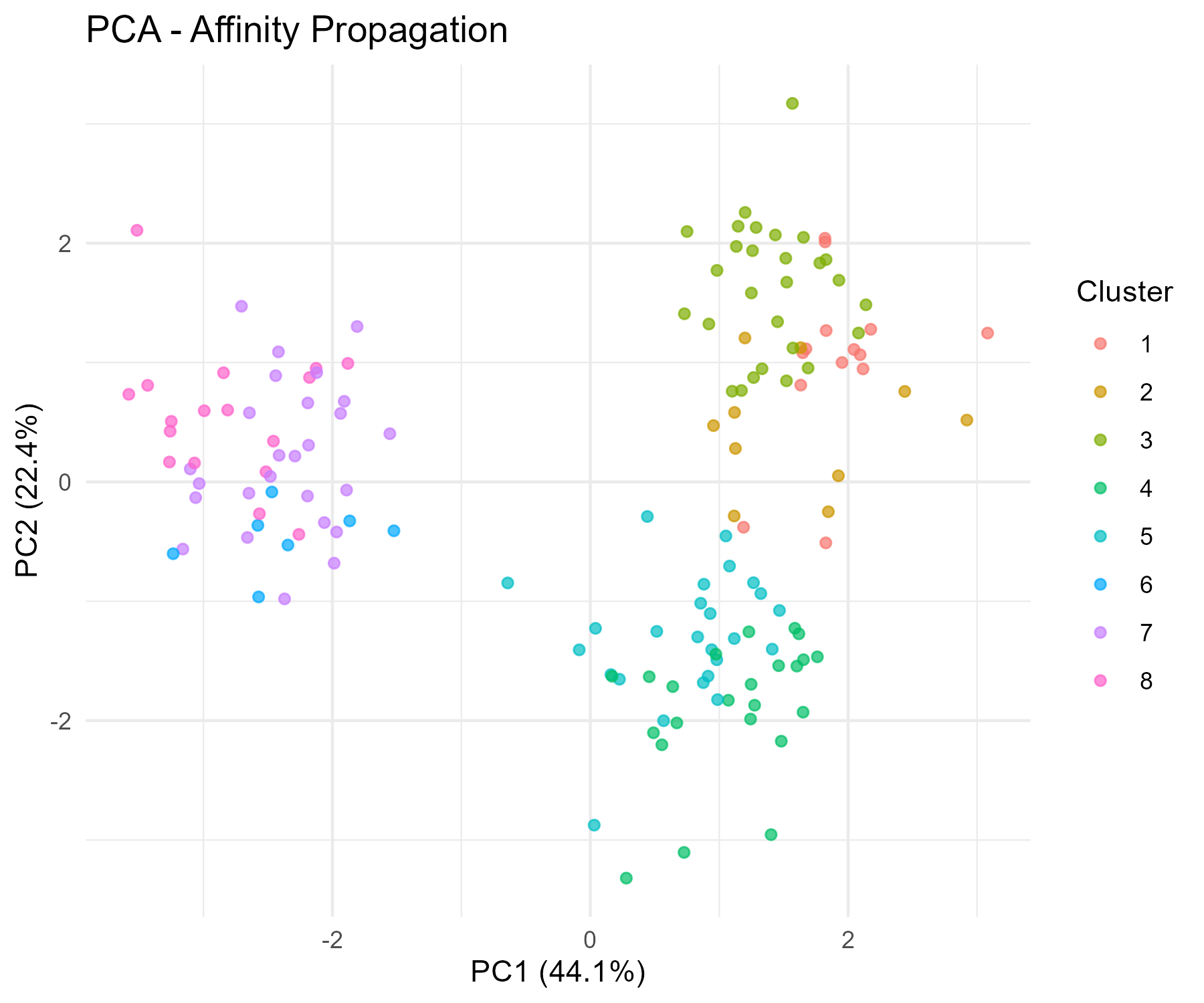

Supplement: S1 File — This compressed archive contains Figures S1–S12 and Table S1. (ZIP) [file pone.0329254.s001.zip › SupportingInformation/S9 (a).png]

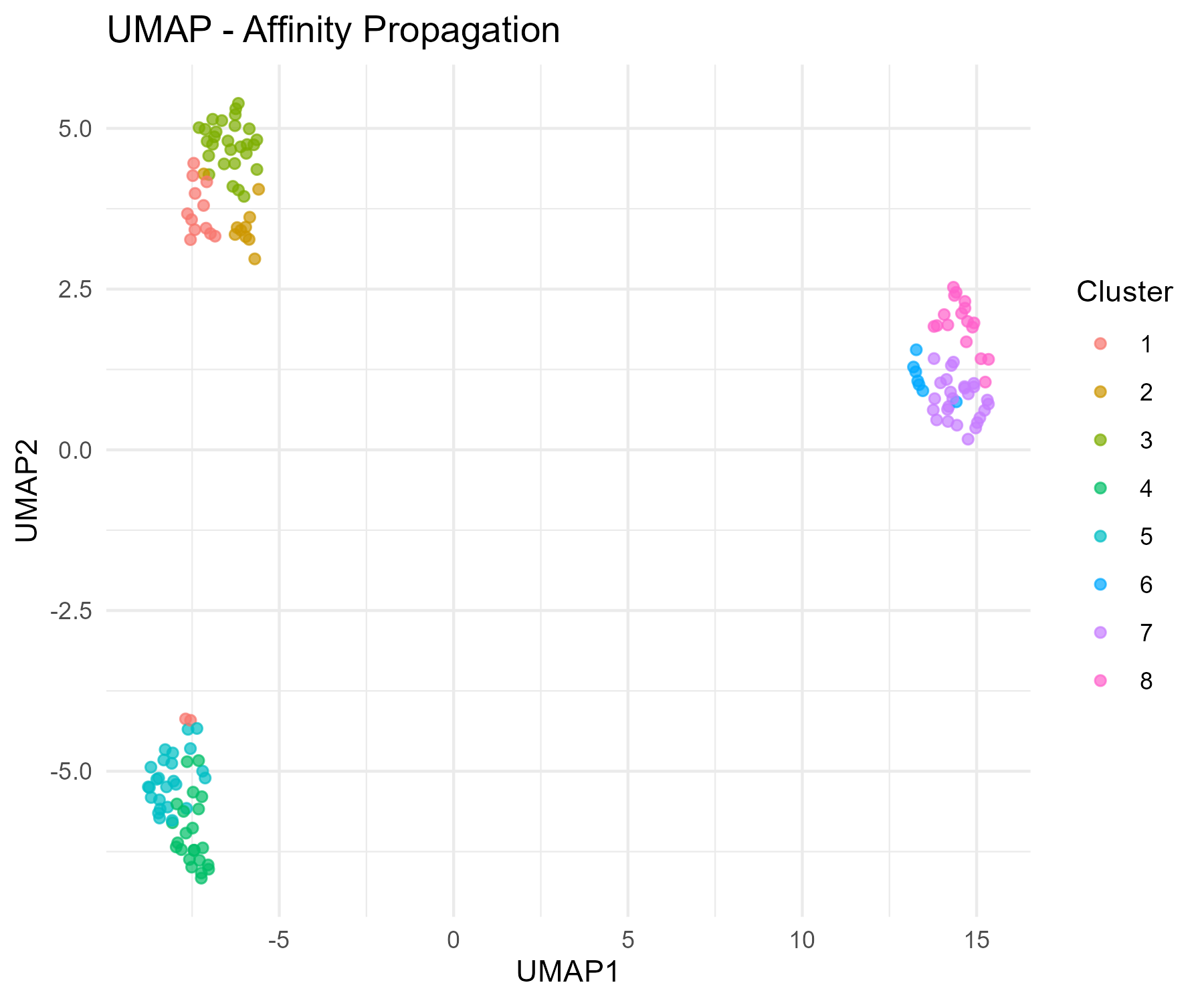

Supplement: S1 File — This compressed archive contains Figures S1–S12 and Table S1. (ZIP) [file pone.0329254.s001.zip › SupportingInformation/S9 (b).png]
