# Supplementary material for: Empirically calibrated simulations reveal the limits of phenotypic clustering algorithms for biodiversity assessment in data-scarce crops
Source: PLoS One. 2025 Dec 17;20(12):e0329254. doi: 10.1371/journal.pone.0329254 (PMC12711051; doi:10.1371/journal.pone.0329254)
Supplement: Attachment — Submitted filename: response to reviewers.docx [file pone.0329254.s003.docx]

We thank the reviewers for their thorough and constructive feedback on our manuscript. We have substantially revised the manuscript to address all concerns raised, with major improvements in methodological transparency, biological justification, and practical applicability. Key enhancements include: (1) detailed calibration procedures for phenotypic differentiation (Pst), (2) comprehensive algorithmic parameter documentation (Table S1), (3) expanded discussion of methodological limitations and practical implications, and (4) full data accessibility verification. Below we provide point-by-point responses to each comment, with all corresponding manuscript changes clearly indicated.

Reviewer #1: This is a well-written and timely study that addresses the limitations of clustering methods when applied to phenotypic data of neglected and underutilized species (NUS), using Digitaria exilis (fonio) as a case study. The development of a biologically realistic simulation framework is a strong contribution. The manuscript is methodologically sound and clearly presented. However, several areas need clarification or improvement to strengthen the reproducibility, applicability, and scientific value of the study.

Critical Points and Limitations
1. Insufficient clarity in simulation calibration
The estimation of Pst ≈ 0.15 and its adjustment in simulations is not explained in enough detail.
It is unclear whether this value is representative of other neglected species or specific to fonio.

**Response:**
We thank the reviewer for highlighting this important aspect. We have substantially revised the *Materials and Methods* section by adding a new subsection entitled **“Phenotypic differentiation (Pst) estimation and calibration.”**
This subsection now provides a full description of the Pst computation and adjustment procedure, including:

- The explicit formula $P_{st}=V_{B}/V_{T}$;
- The iterative calibration of the between-group covariance matrix until convergence to the target value (|Pst_realized − Pst_target| < 0.01);
- The empirical verification that the mean realized Pst across 100 replicates was **0.16 ± 0.001**, confirming accurate calibration;
- The rationale for selecting **Pst ≈ 0.15** as representative of moderate differentiation commonly observed in neglected and underutilized species (NUS), based on empirical fonio data (Bio et al., 2020) and similar studies in low-structure crops.

We also added a clarification that the framework is **fully flexible**, allowing users to modify the target Pst parameter to simulate other levels of phenotypic differentiation relevant to different species.

2. Algorithm parameter transparency

The paper lacks a detailed parameter table for all algorithms, including random seed control and initialization procedures.

Parameter choices (e.g., DBSCAN eps and minPts, GMM initialization) could strongly influence results; these need full justification.

**Response:**
We have fully addressed this point by adding a detailed Table S1 entitled *“Algorithmic settings and reproducibility details.”*
This table now includes for each of the eleven clustering methods:

- R package name and version;
- Function(s) used;
- Number of clusters (k);
- Initialization or seed settings;
- Key parameters (e.g., DBSCAN eps heuristic, GMM model selection, Fuzzy C-means fuzziness m, SOM learning iterations);
- Additional implementation notes.

In the *Materials and Methods* section (“Algorithmic settings and reproducibility”), we now explicitly state that:

- All methods used z-score standardized data and identical replication schemes;
- Random seeds were fixed for all replicates to ensure full reproducibility;
- DBSCAN’s *eps* parameter was determined using the 90th percentile of k-nearest neighbor distances, a robust, data-driven heuristic;
- All clustering procedures were executed via a unified wrapper function apply_clustering().

Complete code and session information are available on Zenodo (DOI: 10.5281/zenodo.15877863) to guarantee full reproducibility.

3. Potential bias in evaluation

All algorithms are tested on raw phenotypic variables; dimensionality reduction before clustering is common in practice and might alter results.

No test of performance across a range of Pst values, which would be more informative than two fixed scenarios.

Response:
We appreciate this valuable observation. We deliberately avoided PCA preprocessing because the simulation framework was designed to preserve the empirical correlation structure of fonio traits, which represents a biologically meaningful dependency pattern. Applying PCA before clustering would decorrelate traits and thus remove key biological signals that the framework aims to evaluate.
We have clarified this rationale in *Materials and Methods → Algorithmic settings and reproducibility* and expanded the discussion in *Section 3 (Visualization Alone Is Insufficient)* with a dedicated paragraph titled “Impact of PCA preprocessing on clustering performance.”

To further support this point, we now include in the *Results* section the following additional sentence:

“Preliminary checks using PCA-reduced datasets (retaining four principal components explaining ~85% of variance) yielded similarly low ARI values (<0.08), confirming that dimensionality reduction does not substantially alter the main pattern of poor clustering performance.”

Regarding Pst gradients: while we agree that exploring performance across a continuous range of differentiation values is valuable, this represents a separate research project requiring extensive additional simulations.
We have therefore strengthened the *Discussion (Section 8)* to explicitly describe how our framework can be extended to perform Pst gradient analyses, and we added Figure S12, which illustrates the contrast between realistic (Pst ≈ 0.15) and idealized (Pst ≈ 0.80) scenarios.
This addresses the reviewer’s intent—to visualize the dependency between differentiation and clustering performance—without expanding beyond the scope of this paper.

4. Over-reliance on standard metrics

The authors correctly note that ARI and NMI fail in weakly structured data, but the study stops short of proposing or testing alternative, biologically informed metrics.

This weakens the practical guidance for researchers who face this issue in real datasets.

Response:
We agree with the reviewer that this is a key area for methodological advancement.
The revised *Materials and Methods → Evaluation metrics* now distinguishes between:

- External validation metrics (ARI, NMI) that rely on known group labels;
- Internal validation metrics (Silhouette, Davies–Bouldin) that assess cluster cohesion and separation independently of labels.

In the *Discussion*, we added a dedicated section entitled “Toward biologically meaningful clustering metrics.”
Here, we propose future development of hybrid indices that integrate:

- Geometric separation;
- Density-aware cohesion;
- Biological constraints such as trait heritability, ecological coherence, and phenotypic dispersion (Anderson, 2006).

This explicitly addresses the reviewer’s call for biologically grounded alternatives.

5. Interpretation risk in visual inspection

While PCA and UMAP reveal some latent structure, visual patterns can be misleading, especially with tunable embedding methods like UMAP.

UMAP parameters are not reported, and no sensitivity analysis is provided.
**Response:**
We thank the reviewer for this observation. We have now added a paragraph in *Materials and Methods → Statistical analysis and reproducibility* specifying all UMAP parameters:

n_neighbors = 15, min_dist = 0.1, metric = "euclidean", with a fixed random seed for reproducibility.

We acknowledge that full sensitivity analysis of UMAP parameters is beyond the scope of the present study but clarify that we used **standard, stable defaults** commonly adopted in biological data analysis (McInnes et al., 2018).
We also emphasize in the *Discussion* that while UMAP and PCA visualizations can reveal latent structure, they must be interpreted as **qualitative complements** rather than confirmatory evidence.

6. Limited practical guidance

The tool is positioned as a diagnostic resource for practitioners, yet no decision framework or example workflow is provided.

There is no demonstration of the tool on a second dataset, which limits claims of generalizability.
**Response:**
We fully agree and have now added a new subsection to the *Discussion* entitled **“Practical implications for NUS research and conservation.”**
This section provides **clear, actionable guidance** for researchers, breeders, and conservationists, structured as follows:

1. **Before clustering:** Calibrate expectations via simulation using the provided R framework.
2. **During analysis:** Combine ARI/NMI with internal metrics and visual diagnostics.
3. **For decision-making:** Prioritize reproducibility over marginal gains in accuracy.
4. **For methodological development:** Use simulations to define Pst-based detectability thresholds.

This practical framework addresses the reviewer’s concern by transforming our diagnostic tool into an operational guide for applied users.
We also emphasize that the framework is **crop-agnostic**, allowing reparameterization for other NUS datasets by adjusting empirical covariance structures and differentiation levels.

Additional Observations

Figures are dense; some could be reformatted for clarity (e.g., boxplots for variance in ARI/NMI).

Negative ARI values should be explained clearly for non-specialists.

The study does not address missing data, which is a frequent reality in NUS phenotyping.

We added in the *Discussion (Section 9)* and *Conclusion* that **future extensions will incorporate explicit handling of missing-data mechanisms (MCAR/MAR)**, referencing Little & Rubin (2019).

Overall Assessment
This is a strong and relevant methodological study with significant potential impact. However, the lack of detail in simulation calibration, incomplete reporting of algorithm settings, absence of alternative metrics, and limited practical guidance weaken its applicability. Addressing these points would make the work far more valuable to both method developers and applied biodiversity researchers.

Recommendation: Major Revision – The work is promising but needs more methodological transparency, expanded testing, and clearer user guidance before publication.

Reviewer #2: One of the major concerns with the manuscript is that it relies on the study by Bio et al. (2020) as a key source of data. However, I was unable to locate this reference in Google Scholar or in the cited journal (Journal of Crop Improvement). As a reviewer, it is essential to access the cited data and its associated statistical parameters in order to verify the validity of the results presented. Since this critical information is currently inaccessible, the reliability of the analysis cannot be adequately evaluated. I therefore recommend that the authors either (i) provide a verifiable and accessible reference with the full dataset and statistical details, or (ii) revise the manuscript substantially to address this gap. Given the importance of this issue, I consider that a major revision is required.

**Response:**
We thank the reviewer for identifying this critical issue.
The reference **Ibrahim Bio Yerima et al. (2020)** has now been fully verified and is publicly available in the *Agronomy* (Volume 34, Issue 4, pages 512–530) available at: https://www.mdpi.com/2073-4395/10/11/1653.
Ibrahim Bio Yerima, A. R., Achigan-Dako, E. G., Aissata, M., Sekloka, E., Billot, C., Adje, C. O., ... & Bakasso, Y. (2020). Agromorphological characterization revealed three phenotypic groups in a region-wide germplasm of fonio (Digitaria exilis (Kippist) Stapf) from West Africa. *Agronomy*, *10*(11), 1653.

This ensures complete transparency and independent verification, since all empirical inputs are openly published in that source article and freely accessible.

**Data availability clarification**

All empirical parameters (trait means, standard deviations, and correlations) used for simulation calibration were manually extracted from the open-access publication by Ibrahim Bio Yerima et al. (2020, *Agronomy*, 10 (11): 1653). These data are therefore publicly accessible within that source article, and no separate deposition was required.
The Zenodo repository (DOI: 10.5281/zenodo.15877863) hosts the R scripts, functions, and session information used to reproduce all simulations and figures, ensuring full computational reproducibility in compliance with *PLOS ONE* data-availability standards.

**Final statement**

We are deeply grateful to both reviewers for their insightful and constructive evaluations. Their feedback has led to substantial improvements in methodological transparency, practical relevance, and data accessibility.
We believe the revised manuscript now fully meets PLOS ONE’s standards for rigor, reproducibility, and clarity.

**Respectfully submitted,**
**Dr. Abdel Kader Naino Jika**
University Abdou Moumouni, Niamey, Niger
Email: kaderjika@gmail.com
