# Supplementary material for: Empirically calibrated simulations reveal the limits of phenotypic clustering algorithms for biodiversity assessment in data-scarce crops
Source: PLoS One. 2025 Dec 17;20(12):e0329254. doi: 10.1371/journal.pone.0329254 (PMC12711051; doi:10.1371/journal.pone.0329254)
Supplement: Attachment — Submitted filename: Response_to_Reviewers_auresp_2.docx [file pone.0329254.s004.docx]

**Manuscript ID:** PONE-D-25-38295R1
**Title:** *Empirically calibrated simulations reveal the limits of phenotypic clustering algorithms for biodiversity assessment in data-scarce crops*

**Dear Academic Editor and Reviewer #1,**

We sincerely thank you for your constructive and positive evaluation of our revised manuscript. We are grateful for the reviewer’s detailed comments, which have helped further improve the clarity and presentation of the work.

Below we provide a concise, point-by-point response describing the changes implemented in the final revised version.

**Reviewer #1 Comment 1 — Clarity on Negative ARI**

*“The Discussion and results should briefly clarify the interpretation of negative ARI values.”*

**Response:**
We thank the reviewer for this important suggestion.
We have clarified the interpretation of negative ARI values in both the *Results* and *Discussion* sections:

- **Results (Section: Clustering performance under realistic conditions):**
  We revised the sentence to improve clarity and accessibility for non-specialist readers:

“Negative ARI values indicate that agreement between predicted and true partitions is slightly lower than would be expected by chance. This occurs when clusters are statistically misaligned under weak phenotypic differentiation, and should not be interpreted as algorithmic failure but rather a signal of diffuse or overlapping biological structure.”

- **Discussion (Point 1 – Fundamental methodological mismatch):**
  We added the statement suggested by the reviewer:

“The small negative ARI values observed for some algorithms further confirm that, under these weakly structured conditions, agreement with the true partition can fall below what would be expected by chance alone.”

These additions directly address the reviewer’s request and improve interpretability for non-specialist readers.

**Reviewer #1 Comment 2 — Figure density and readability**

*“Ensure the legends and labels are optimally clear and legible in the final production format, particularly for the multi-panel composites.”*

**Response:**
We carefully revised the multi-panel composite figure (Figure 3) by increasing the font size of axis labels, legend text.
No scientific content was altered; only graphical readability was improved.
The revised figure now meets PLOS ONE’s visibility requirements at ≥ 300 ppi.

**Additional minor editorial improvements**

In accordance with PLOS ONE guidelines, we made the following minor corrections throughout the manuscript:

- Standardized section and subsection titles using **sentence case** (capital letter only at beginning).
- Corrected minor typographical inconsistencies.

These revisions do not change the scientific content.

**Final Statement**

We thank the reviewer and the Academic Editor for their thoughtful feedback and appreciation of the methodological rigor of this work. We believe these revisions fully address the remaining minor concerns. We remain at your disposal for any additional clarification.

Sincerely,
Abdel Kader NAINO JIKA
